# Chemical Analysis of the Urine in a Case of Diabetes Mellitus

**Published:** 1804-02-01

**Authors:** 

**Affiliations:** Berlin


CHEMICAL ANALYSIS *of the* URINE *in a* CASE OF DIABETES MELLITUS, *by Professor* KLAPROTH, *of Berlin.*

I Had lately an opportunity of examining the urine of a patient, labouring under diabetes mellitus, which was transmitted to me by Dr. Michaelis, of Harburg. The colour of this liquor was pale yellow; and a small portion of a reddish sediment, had fallen to the bottom of the glass in which it it was contained. The smell was neither putrid nor ammoniacal, but the taste, as well its changing the blue vegetable colour to a red, evidently betrayed the traces of its containing an acid, which I thought had been generated by the saccharine matter passing into acetous acid; an alteration which it was likely to have undergone, as it had been kept above six months in a warm place. Besides this acid, I discovered in the liquor evident traces of phosphoric acid, for on being mixed with acetite of lead it precipitated a white substance, part of which could be reduced to lead, while another part was melted to a white porcelain globule, which on cooling received a polyedrical form, being thus characterised as phosphate of lead. The reddish sediment was accurately examined for the purpose of finding in it the acidum urolithicum, of which, however, not the least trace could be perceived, it being found chiefly to consist of albuminous matter.

With a small quantity of the above urine, &c. Dr. M. had sent me a portion of the residuum of the same diabetical urine, after its having been evaporated to the consistency of honey. It had the appearance, the blackish colour, and consistency of a thin extract, and contained a sediment of small brown and spherical crystals; it had a sourish taste; no ammonia could be disengaged by mixing it with caustic kali, and it remained without any smell. On adding to it carbonated kali, a considerable effervescence took place. Being infused with four times its measure of alcohol, and digested with a very moderate heat, the greatest part of it was dissolved except a gummous residuum and the above mentioned salt. After this solution had been separated from the residuum through the filtrum, and evaporated to the consistency of an extract, it was mixed with three times its measure of moderately strong nitric acid, whereby a strong reaction ensued in the heat, a considerable quantity of nitrous gas being at the same time disengaged. The residuum appeared of the colour and consistency of pure honey, which being again treated with the same quantity of nitric acid, was for the most part changed into oxalic acid, shooting into crystals on cooling. The saline and gummous residuum, which remained insoluble in alcohol, was dissolved in hot water, which being cooled, deposited the salt in form of light brown crystals, which seemed to be flat tetraedical prisms. As I had discovered in the urine the presence of phosphoric acid, I concluded those crystals might also contain it, in which however I was disappointed. They easily melted on a coal a coal before the tubus fusorius, bnt formed no pearl, leaving behind a fixed alkaline salt, which being strongly heated in a platina crucible, dissolved in water, and supersaturated with a solution of tartaric acid, yielded pure tartar. They made a strong precipitation in lime water, but the sediment being heated proved to be only caustic lime, which was soluble in water. In a solution of acetite of lead they precipitated a white substance, which was entirely reduced to lead.

From these and similar experiments these crystals appeared to consist of nothing but oxalat of kali. Alcohol precipitated the gummous particles from the watery solution in form of a light brown glutinous substance. The inspissated urine resembled more a vegetable extract than an animal substance; but particularly, the specific constituent of the urine, the ureum, was not contained in it. The results of these experiments agree with those which Dr. Rollo, Mr. Cruikshank, and lately Messrs. Nicolas and Gneudeville have communicated, except that these gentlemen having examined fresh urine, found the chief constituent of it to consist of mucosaccharine matter, which in the urine I examined had been changed into an acid extractive matter. It is remarkable, that no phosphoric acid could be traced in the inspissated urine, which I had found in the raw urine. The above extract of urine, mixed with acetite of lead, yielded a great quantity of an ash coloured sediment, which was easily reduced to metallic lead.

